# The Shift to Over-the-Counter Diagnostic Testing After RADx: Clinical, Regulatory, and Societal Implications

**DOI:** 10.1109/OJEMB.2024.3512189

**Published:** 2024-12-09

**Authors:** Maren Downing, John Broach, Wilbur Lam, Yukari C. Manabe, Greg Martin, David McManus, Robert Murphy, Apurv Soni, Steven Schachter

**Affiliations:** ^1^ M Biomedical LLC McCormick SC 29835 USA; ^2^ School of MedicineUMass Chan 12262 Worcester MA 01655 USA; ^3^ Department of Pediatrics and the Wallace H. Coulter Department of Biomedical EngineeringEmory University School of Medicine and Georgia Institute of Technology 209740 Atlanta GA 30307 USA; ^4^ Division of Infectious Diseases, Department of MedicineJohns Hopkins University School of Medicine 1500 Baltimore MD 21205 USA; ^5^ Division of Pulmonary, Allergy, Critical Care and Sleep MedicineEmory University School of Medicine 12239 Atlanta GA 30307 USA; ^6^ Division of Cardiology, Department of MedicineUMAss Chan Medical Scool 12262 Worcester MA 01655 USA; ^7^ Program in Digital Medicine, Department of MedicineUMass Chan Medical School 12262 Worcester MA 01655 USA; ^8^ Havey Institute for Global HealthNorthwestern University 12244 Chicago IL 60611 USA; ^9^ Division of Health System ScienceUMass Chan Medical School 12262 Worcester MA 01655 USA; ^10^ Center for Integration of Medicine and Innovative Technology Boston MA 02114 USA; ^11^ Department of NeurologyMassachusetts General Hospital 2348 Boston MA 02114 USA; ^12^ Department of NeurologyBeth Israel Deaconess Medical Center and Harvard Medical School 207096 Boston MA 02215 USA

**Keywords:** Diagnostics, in vitro diagnostics, OTC, point of care, viral testing

## Abstract

The National Institutes of Health's Rapid Acceleration of Diagnostics (RADx) program answered the call to accelerate the development of point-of-care (POC) and over-the-counter (OTC) COVID-19 tests. The widespread availability and access to self-tests has increased the public's familiarity and acceptance of at-home diagnostics. This paper examines the current state of OTC diagnostic testing, discusses potential applications of OTC testing, and highlights the implications of widespread OTC testing for clinical medicine.

## I. Introduction

The COVID-19 pandemic forced the world at large to function predominantly from home. Coupled with medical facilities being stretched to capacity, non-emergent medical traffic decreased and catalyzed an exponential rise in self-testing. Congress recognized the urgent need for rapid point-of-care (POC) diagnostics during the pandemic, and other agencies such as the U.S. Food and Drug Administration supported this effort by releasing a guidance for Emergency Use Authorizations on over-the-counter (OTC) self-tests that are performed and interpreted by the individual.

The National Institutes of Health's Rapid Acceleration of Diagnostics (RADx) Program answered the call for COVID-19 OTC test development, developing platforms that delivered

7.8 Billion tests beginning in 2020 to 2023 [1], [2], [3], [4]. This newfound availability of home-based tests has since increased the public's acceptance and use of home diagnostics. The demand for OTC tests will continue to grow for respiratory viruses, including influenza and respiratory syncytial virus, and at present, efforts have turned to developing multiplex testing panels. If individuals are interested in diagnostic certainty and self-tests, these multiplex platforms could pivot to address other syndromes with different sample types. (e.g., diarrhea, bloodborne infectious viral hepatitis, or HIV). This calls for greater evidence generation to support multiplex testing use amongst home users [5].

OTC diagnostic testing is important for reducing disease transmission, chronic disease management, and potentially access to healthcare. In situations such as pandemics, having tests that can quickly identify individuals carrying the virus could help to prevent outbreaks and reduce disease transmission [6]. Putting testing power in the hands of the patient can allow for earlier disease detection or potentially delay the onset of disease. Many diseases require early intervention after diagnosis. In the case of pharyngitis due to Group A Streptococcus (GAS), early diagnosis is important for antibiotic initiation to impact the course of care and to prevent disease sequelae like acute rheumatic fever [4]. Allowing for testing at home can decrease disease burden, for example, by preventing infectious individuals from entering public spaces. Also, regular monitoring of chronic diseases, such as diabetes mellitus, can allow for better disease control and therefore complication prevention and potential decrease in disease burden. OTC tests can allow for timely decision making for people living in rural areas who may struggle with access to care for geographic reasons. In addition, it makes obtaining a diagnosis for uninsured or underinsured persons more affordable assuming that the test itself is affordable.

Even with the boom of OTC testing for COVID-19, there are challenges to product development and end-user adoption. Here, we discuss the adoption of OTC tests from the regulatory, societal, and clinical perspectives, and explore potential implications of widespread OTC testing on clinical medicine.

## II. Pre-RADx Landscape for Home-Based Tests

Long-term market size was a pre-pandemic concern for developers; and for manufacturers, the value of the proposed solution must be clear with a sufficient target market for profitability after commercialization. Historically, these development challenges have stifled innovation in the OTC testing arena. The potential for user error presents major challenges for developers, testing procedures are not always widely accepted, and there are inevitable technology performance limitations and trade-offs (i.e., specificity vs sensitivity). There is a hurdle to communicating the clinical meaning of a test with lower sensitivity to a home user. And, in some cases, there may be additional hurdles imposed by regulatory authorities for approvals in the form of cost and time, and special controls–HIV screening self-tests are classified as class III due to increased risk to the individual if a test is incorrectly interpreted; this designation incurs significant development and regulatory approval costs compared to a class II device [8]. The first HIV self-test, OraQuick (OraSure Technologies, Bethlehem, PA USA) was approved by the FDA in 2012 with no subsequent OTC HIV tests despite significant improvements in the sensitivity. Compared to POC, CLIA-waived diagnostics meant to be performed in clinical settings, regulatory pathways were not well-established for OTC testing. For some communicable diseases such as gonorrhea, chlamydia, HIV, and viral hepatitis, OTC testing could have important public health impact, yet regulatory pathways are a one size fits all approach.

## III. The Home Testing Surge and Its Advantages

Researchers are developing OTC tests for other infectious diseases such as GAS and influenza, common causes of trips to the doctor's office. An estimated 5.2 million outpatient visits are conducted annually for strep throat in the United States [9]. The FDA granted Emergency Use Authorization to a combined SARS-CoV-2 and influenza OTC test in February 2023 [10].

The push for at-home testing for sexually transmitted infections (STIs) was accelerated by the COVID-19 pandemic [11]. It was estimated that between 16 million and 19 million tests were performed in the year 2016 for *C. trachomatis* and *N. gonorrhoeae*, respectively, two sexually transmitted infections among the top 10 U.S. notifiable conditions with screening recommendations [11]. There are existing POC, CLIA-waived rapid tests for gonorrhea, chlamydia, and trichomonas, and screening tests for syphilis [12]. However, in the case of STIs, there are varying state laws, with some states allowing only medical professionals to order diagnostic testing, which poses a hurdle for widespread acceptance.

As was the case with at-home HIV testing, patients recognize the benefits of self-testing including privacy, convenience, and ease of use [12]. Patients are empowered to take action in their own healthcare and gain autonomy through self-testing. A person's health is a highly personal matter and conducting a test in the comfort of their own home may be more appealing for sensitive conditions (i.e., STIs). Fear may lessen a person's willingness to see a doctor, preventing those in need from getting appropriate treatment. The emergence of OTC tests is congruent with the concept of self-efficacy, which holds that patients believe in their own abilities to make appropriate decisions for themselves based on their knowledge of results.

Decreasing the number of infectious persons entering public areas like health clinics promotes population health benefits. Decentralizing POC diagnostic testing in medical office settings to the home could theoretically enhance the provider experience along with that of the patient. Health clinic personnel could save time that would otherwise be spent awaiting and communicating test results. Depending on the medical practice, respiratory virus testing may not be available in the office unless there are severe comorbidities or if the patient is immunocompromised. Therefore, this raises the question of the public's appetite for diagnostic certainty along with cost-sensitivity.

## IV. Challenges of Widespread OTC Testing to Address

### A. Clinical

A shift to OTC testing provides patients with greater access to care through saved time, possibly lower costs, and enhanced virtual appointments. Timely access to patient care appointments is crucial, and many patients end up using urgent care or the emergency department if their primary care provider has an extensive wait time for an appointment [13]. If affordable, a user with symptoms can elect to do a self-test and potentially avoid a trip to the doctor's office, thereby freeing up time for more appointments for the provider. Healthcare costs can be saved through the appointment itself but also the transportation to that appointment and missed time from work. With the booming popularity of telemedicine today, being able to make a diagnosis from the home would be a profound addition to the efficacy of a virtual visit. However, clinicians would need to accept the results of home-based OTC tests and give appropriate prescriptions accordingly without repeating the test.

OTC testing allows patients to participate in health testing without necessarily being under the direction of a clinical provider. In the absence of a clinician, home-testing users may not receive educated direction on what their test result means. Therefore, a consumer may misinterpret test results or act inappropriately to a received result, posing a potential health risk to the individual. Health literacy becomes a concern, as patients may not know the clinical meaning of a positive or negative result and which actions to take based on their result. Clinicians assess the history of the present illness and associated symptoms along with the patient's pertinent medical history to advise on what diagnostic testing, treatment, or specialty evaluations are needed. They interpret and explain results to the patient in the context of their patient's own history. There is less information or patient data collected when tests are done at home, and this may not leave healthcare professionals with enough data to make evidence-based decisions [14]. A patient's established relationship with their primary care provider is crucial for promoting health—further research on the impact of the provider-patient relationship and retention is needed.

There is no control on patient appropriateness for OTC test taking [15]. Not all medical testing is appropriate for preventative care. It should be up to the clinician and the patient to determine what is appropriate. Individuals with no medical background obtaining results of medical tests at home may be compelled to act on those results, when not every abnormal lab value requires an action. This could cause undue worry to an individual, and raises the concern of how much knowledge is too much knowledge? If a patient took a test for the wrong reasons, and ended up with an actionable result, then a clinician may want to retest the patient with a laboratory test anyways. Having a test available at home could generate anxiety for users but also give them false reassurance. OTC tests that require a provider prescription mitigates this risk, but also decreases access for individuals without a provider.

### B. Performance

Aside from social challenges, there are performance hurdles for tests intended for OTC use. Acute infection with HIV has a ‘window’ during which an individual would test negative by antigen antibody tests, but have a detectable HIV viral load [16]. Serologic testing for hepatitis C is challenging as a person with past HCV infection that has cleared with be serologically positive, but not require treatment. Similarly, for syphilis screening, anti-treponemal antibody tests are positive for life regardless of treatment. A secondary confirmatory test is required to be actionable. All IVD tests have inherent limitations on sensitivity and specificity whether POC or OTC, and test performance must be maintained during at-home testing. This must be balanced against access to testing in vulnerable populations.

In the case of COVID-19 OTC testing, many at-home test kits have lower sensitivity than PCR tests and a negative at-home result does not necessarily mean the user is not infectious. Follow-up testing is recommended, but a randomized clinical trial assessing how Americans interpret at-home COVID-19 self-test kits found that a substantial percentage of those studied (33%) with a high pretest probability of infection misinterpreted the implications of their negative results and proceeded to ignore quarantine recommendations [17].

Pretest probability can depend on variables such as disease prevalence, exposure history, and symptoms [18], all factors that a clinician would consider in a patient evaluation but that an at-home user may not take into account. A low prevalence of infection with SARS-CoV-2 will lead to false positive results when testing is done broadly [6]. This would then require further testing for confirmation.

### C. Regulatory

A commonly known strategy for avoiding the regulatory approval process in the United States is to indicate that a test only provides healthcare information, not a diagnosis [7]. However, if a patient utilizes an at-home test only for information purposes, it is likely that they would expect their healthcare provider to use those results for medical decision-making. This makes it difficult to place tests in the hands of consumers as they will not distinguish between obtaining a result only for information or for a diagnosis.

Additionally, it is impossible to verify the identity of the test taker [19]. This becomes important when reporting is required for notifiable diseases. The COVID-19 Citizen Science Study found that at-home tests comprised greater than 80% of all SARS-CoV-2 testing reported, and that positivity trends were starting to diverge between those numbers and that of national data from reported tests [20]. Inconsistencies in reporting may underestimate a number of infectious individuals, resulting in potential public health concerns. For OTC COVID-19 testing, MakeMyTestCount was developed as an effort for self-reporting of results [21]. The use of telemedicine could also combat nonreporting issues as results would be communicated with a medical provider through that virtual interaction.

There are no regulations on who may or may not be using an at-home test, whereas prescription tests have oversight by healthcare workers determining the intended use population. Test stability and shelf life may not be monitored by the end users, allowing expired tests to still be used by consumers, which can result in inaccurate test results.

### D. Design

Common mistakes in at-home testing include failing to prepare test kits correctly, specimen collection challenges, and spilling buffer solutions [12]. This can be mitigated with adequate instructions, but that requires that the end user be able to understand and interpret instructions and labeling. Testing workflow must not be too cumbersome and the number of steps must be considered when designing a test to be performed at home by a nonprofessional. The turnaround time for results must be within a reasonable range for any test with a POC or OTC designation, taking into account the urgency of receiving the results. Laypersons with no prior experience or training and those with disabilities, will be testing in non-clinical settings where no supervision or aid is offered [5]. Accessibility should also be considered for OTC assays.

Properly collected specimens are crucial for accurate results. A poorly collected specimen can lead to false-negative results. A shift to OTC testing, especially for respiratory pathogens, will require further research of additional sampling methods as laypersons may not accurately self-sample. Researchers have raised concerns about specimen collection for GAS [7]. For specimens that may be difficult to obtain, as in the case of STIs, remote providers can instruct patients on how to do so [11]. Specimen integrity also remains an issue, such as temperature, handling, or delay in transit [11].

## V. Conclusion

The widespread acceptance of OTC tests during the COVID-19 pandemic makes future development and commercialization of other OTC diagnostic tests promising. The resulting impact of the RADx program will allow for continued proliferation of tests in the future for further applications. This is a big space for innovation, but developers must consider end-user adoption for design. OTC popularity coupled with the predicted future direction of healthcare towards non-traditional settings further underscores the increasing importance of home-based testing options for many medical conditions.

## Conflicts of Interest

MD has received financial support from VentureWell during the writing of this manuscript. YM has received research grant support to Jons Hopkins University from Hologic, Cepheid, Roche, and ChemBio. DDM reports consulting and research grants from Bristol-Myers Squibb and Pfizer, consulting and research support from Fitbit, consulting and research support from Flexcon, research grant from Boehringer Ingelheim, consulting from Avania, non-financial research support from Apple Computer, consulting fees from NAMSA, consulting fees from VentureWell, and consulting/other support from Heart Rhythym Society. AS received consulting fees from ARPA-H, Pfizer, and AAMC for work unrelated to this manuscript.

## Author Contributions

All authors made a significant intellectual contribution to the development of this manuscript and contributed to drafting, reviewing, or revising it for intellectual content and approved the final version including references.
